# Editorial: Self-regulated learning in online settings

**DOI:** 10.3389/fpsyg.2022.968586

**Published:** 2022-08-01

**Authors:** Paula Galvao De Barba, Jaclyn Broadbent, Danial Hooshyar, Erin Peters-Burton

**Affiliations:** ^1^The University of Melbourne, Parkville, VIC, Australia; ^2^Deakin University, Geelong, VIC, Australia; ^3^Tallinn University, Tallinn, Estonia; ^4^George Mason University, Fairfax, VA, United States

**Keywords:** self-regulated learning, online learning, editorial, metacognition, educational technology (EdTech)

Online learning has become an increasingly popular mode of learning in today's education. With the COVID-19 pandemic resulting in stay-at-home orders worldwide, most, if not all, students will have experienced online learning to some degree. Given the high level of autonomy required with online learning, self-regulated learning (SRL) is essential for academic success when studying online (Broadbent and Poon, 2015). SRL refers to the various ways individuals plan, monitor, control, and regulate their learning, with most SRL frameworks containing three phases: preparatory, performance, and appraisal (Zimmerman, 2008).

Even though research in SRL has spanned over 50 years, online SRL is still an emerging field. The 13 selected articles for this special issue focused on online SRL. They involved 57 authors from 17 countries (Australia, Canada, China, Czechia, Estonia, Finland, France, Germany, Liechtenstein, Malaysia, Netherlands, Norway, Poland, Slovakia, Spain, United Kingdom, and the United States). Five papers focused on better understanding online SRL, and eight focused on examining interventions to support online SRL. These are outlined below.

Because of the ability to gather, synthesize and analyse huge amounts of data, technology is an avenue to examine the ways SRL strategies are used. Taub et al. investigated how students' SRL behavior and achievement-goal orientation changes over one semester. The authors highlighted the changing nature of SRL during online learning as a series of SRL events that unfold and provide guidance for developing online instructional materials that facilitate SRL for students with different motivational profiles.

In a similar effort to use technology to understand SRL, Lim et al. employed a pre-post design to measure students' SRL in an online learning environment via concurrent think-aloud protocols. Aside from identifying how students of varying success use regulation processes during learning, their study obtained evidence of relations between SRL activities and transfer performance. The authors conclude that interventions should focus on a repertoire of SRL strategies and knowing when to use them.

Non-academic learning settings, such as sports, continue to provide insights into understanding and generating new ways of supporting SRL in academic learning settings. Kleinman et al. replicated Kitsantas and Zimmerman's (2002) original volleyball microanalytical study examining the use of SRL in the online context of esports. When comparing the use of SRL processes between experts, non-experts and novices, they found that novices struggled only in the forethought phase. The authors discussed how specific features of esports, such as visualization of their cumulative data, could support novices during the performance and self-reflection phases.

Investigations on SRL during the COVID-19 pandemic showed that such disruptive events affect students' ability to perform well. Still, effective SRL interventions can serve as a buffer for these additional challenges. Kilmova et al. studied undergraduate students' online SRL during the sudden and seismic shift to online learning. They found that students reported positively on motivation, personal competencies and meaningfulness. However, students struggled with metacognitive strategies. They suggested that instructors gradually introduce SRL strategies to their students explicitly, underscoring the need to know the interaction of learning through technology and SRL in a more detailed way.

Similarly, Hadwin et al. examined whether SRL practices and intervention helped post-secondary students mitigate the impact of COVID-related psychological distress and academic challenges on their educational outcomes. They surveyed 463 students at the end of a semester on their SRL practices and then compared first-year undergraduate students who did and did not participate in an SRL intervention (a 13-weeks course on learning strategies). Findings showed that SRL practices buffered the impact of COVID challenges on students' academic performance and that an intervention can effectively support these practices.

On SRL interventions, Edisherashvili et al. conducted a systematic review on initiatives to support SRL in online higher education settings. Across the 38 studies considered, they found various effective SRL interventions, particularly in metacognitive regulation and the performance phase of learning. They found a lack of SRL interventions in emotion regulation and across the preparatory and appraisal phases of SRL.

The effectiveness of technology-based SRL interventions was tested across four papers. Two studies by Baars explored the efficacy of a mobile application called Ace Your Self-Study, which supports SRL. The first article by Baars Zafar et al. describes the design and development of the mobile application, including theoretical background, app features, embedding gamification elements and targeting all three phases of SRL. The second article by Baars et al. focused on implementing the Ace Your Self-Study mobile application in a first-year psychology course. Compared to students not using the application, students using the application had a significant increase in autonomous motivation, controlled motivation, and metacognitive self-regulation skills across the 5-week course. Qualitative interviews provided additional complementary insights into the mobile application's efficacy.

Additionally, Han et al. used a quasi-experimental design to examine undergraduate students perceived SRL strategies in three writing task conditions: two technology-enhanced groups (Icourse and Icourse + Pigai) and a control group. They found that the two technology-enhanced groups significantly outperformed the control group but performed similarly to one another. This study adds to the evidence that technologically mediated learning can support SRL and demonstrates the need to dig deeper into why and how technology can support SRL. Bellhäuser et al. conducted a randomized controlled trial to investigate the effectiveness of different web-based SRL interventions (training, learning diary and peer feedback) on improving SRL knowledge, self-efficacy, time investment and content acquisition. Web-based training was an effective intervention to improve all outcomes, except content acquisition, especially when combined with peer feedback groups. Learning diaries, on the other hand, did not affect measured outcomes.

However, as Deter et al. and Azevedo et al. pointed out in their papers, SRL is complex and requires new conceptual and methodological approaches, such as considering SRL as a complex system. This shift would allow the upcoming generation of adaptive and personalized interventions to grasp the dynamic and emergent nature of SRL fully. Dever et al. described an experiment in which the learner's navigation through a game-based learning environment was manipulated, i.e., full agency and partial agency. The Authors found that learners with restricted agency and more recurrent actions had greater learning gains. Azevedo et al. offer lessons learned and future directions for MetaTutor, an intelligent tutoring system that provides SRL-based scaffolds in the context of learning about the human circulatory system. Through studies involving MetaTutor over the past 10 years, the group has gathered an enormous amount of information on the role of cognitive strategies, metacognition, emotion, and motivation while learning with the intelligent tutoring system. They offer ideas and limitations for studying human and artificial agents and emphasize the interdisciplinarity needed for thorough examinations of SRL.

Finally, Khalil and Belokrys analyzed the online public discussion of SRL through 54,070 tweets and 29,556 users' over the last 10 years via Twitter. The authors found five overarching themes of what people were discussing about SRL online: communication and help-seeking, self-control, mindfulness, online workshops, and assessment. This paper provides valuable insights into the online public discourse of SRL and how to enhance SRL research impact.

These articles reflect the current need to keep understanding SRL as sequences of events unfolding overtime in online settings. Moreover, they call for finding innovative and effective ways to provide students with SRL support, e.g., personalized scaffolding of SRL in real-time. We hope this collection inspires upcoming SRL researchers to continue to explore SRL in online settings.

## Author contributions

PG wrote the first draft of the manuscript. All authors contributed to writing summaries of the papers, manuscript revision, read, and approved the submitted version.

## Conflict of interest

The authors declare that the research was conducted in the absence of any commercial or financial relationships that could be construed as a potential conflict of interest.

## Publisher's note

All claims expressed in this article are solely those of the authors and do not necessarily represent those of their affiliated organizations, or those of the publisher, the editors and the reviewers. Any product that may be evaluated in this article, or claim that may be made by its manufacturer, is not guaranteed or endorsed by the publisher.
